# Clinical Utility of a Plasma Protein Classifier for Indeterminate Lung Nodules

**DOI:** 10.1007/s00408-015-9800-0

**Published:** 2015-09-16

**Authors:** Anil Vachani, Zane Hammoud, Steven Springmeyer, Neri Cohen, Dao Nguyen, Christina Williamson, Sandra Starnes, Stephen Hunsucker, Scott Law, Xiao-Jun Li, Alexander Porter, Paul Kearney

**Affiliations:** Pulmonary, Allergy, and Critical Care Division, Perelman School of Medicine, University of Pennsylvania/Abramson Research Center, 3615 Civic Center Boulevard, Suite 1016E, Philadelphia, PA 19104 USA; Henry Ford Hospital, 2799 W. Grand Blvd., Detroit, MI 48202 USA; Greater Baltimore Medical Center, 6569 North Charles Street, Suite 701, Baltimore, MD 21204 USA; Sylvester Comprehensive Cancer Center, University of Miami Hospital & Clinics, 1550 NW 10th Avenue, Fox Building, Suite 308, Office 314, Miami, FL 33136 USA; Department of Thoracic and Cardiovascular Surgery, Lahey Hospital & Medical Center, 41 Mall Road, Burlington, MA 01805 USA; University of Cincinnati, 231 Albert Sabin Way, Cincinnati, OH 45267-0562 USA; Integrated Diagnostics, 818 Stewart St., Suite 1101, Seattle, WA 98101 USA

**Keywords:** Xpresys lung, Lung nodule, Clinical utility, Prospective, Biomarker, Lung cancer

## Abstract

Evaluation of indeterminate pulmonary nodules is a complex challenge. Most are benign but frequently undergo invasive and costly procedures to rule out malignancy. A plasma protein classifier was developed that identifies likely benign nodules that can be triaged to CT surveillance to avoid unnecessary invasive procedures. The clinical utility of this classifier was assessed in a prospective–retrospective analysis of a study enrolling 475 patients with nodules 8–30 mm in diameter who had an invasive procedure to confirm diagnosis at 12 sites. Using this classifier, 32.0 % (CI 19.5–46.7) of surgeries and 31.8 % (CI 20.9–44.4) of invasive procedures (biopsy and/or surgery) on benign nodules could have been avoided. Patients with malignancy triaged to CT surveillance by the classifier would have been 24.0 % (CI 19.2–29.4). This rate is similar to that described in clinical practices (24.5 % CI 16.2–34.4). This study demonstrates the clinical utility of a non-invasive blood test for pulmonary nodules.

**Electronic supplementary material:**

The online version of this article (doi:10.1007/s00408-015-9800-0) contains supplementary material, which is available to authorized users.

## Introduction

A large number of pulmonary nodules are identified annually in the U.S. presenting a difficult clinical challenge, as the majority ultimately prove to be of benign origin [1]. Physicians are faced with developing a diagnostic strategy that identifies nodules that are malignant and yet minimizes the risks of invasive procedures on benign nodules. Currently available tools to assist physicians include risk prediction models [2] and imaging, such as PET/CT. However, evidence suggests that these tools have limitations in practice [3, 4].

With the adoption of low-dose CT (LDCT) screening for lung cancer [5] and the anticipated increase in pulmonary nodules, there is a growing interest in biomarkers as diagnostic adjuncts [6, 7]. Advances in proteomic technologies permitted the development of a 371 protein classifier composed of lung cancer-associated proteins [6]. The discovery phase identified a subset of five diagnostic proteins that participate in several lung cancer-associated pathways (pathogenesis, chronic lung inflammation, oxidative stress response) [6]. The classifier was further validated in patients with indeterminate pulmonary nodules to identify likely benign nodules with high accuracy (negative predictive value 90 %, sensitivity 92 % and specificity 20 %). The classifier is additive to currently used clinical risk factors, and has high analytic performance [8, 9]. The intent of the present study is to evaluate the clinical utility of this classifier in a multicenter study of patients with indeterminate nodules undergoing invasive diagnostic procedures.

## Methods

### Study Design and Population

The study used a prospective-specimen-collection and retrospective-blinded-evaluation (prospective–retrospective) design. Patients with an indeterminate pulmonary nodule were enrolled at 12 geographically diverse sites in the U.S. Eligible patients were those with a lung nodule between 8 and 30 mm in diameter, minimum 40 years of age, and had recently completed a CT-guided needle aspiration (TTNA) or bronchoscopic biopsy with an established diagnosis or scheduled for a surgical lung biopsy. Exclusion criteria included a prior malignancy within 5 years of lung nodule identification or a clinical tumor stage ≥T2, nodal stage ≥N2, or evidence of metastatic disease.

All TTNA and bronchoscopy procedures were categorized as either diagnostic (provided a specific malignant or benign pathological diagnosis), or non-diagnostic (the specific etiology of the lung nodule remained unknown). All surgical procedures were categorized into either diagnostic (i.e., no specific prior diagnosis) or therapeutic (i.e., surgery preceded by a TTNA or bronchoscopy that yielded a malignant diagnosis).

For comparison to practice patterns, we utilized a retrospective-observational study of 377 patients with pulmonary nodules between 8 and 20 mm, presenting to 18 geographically representative outpatient pulmonary clinics [3, 4]. In addition to the published data for the study, an additional analysis was performed to determine the number of non-small cell lung cancer (NSCLC) patients triaged to CT surveillance during management, specifically, those having a second CT scan at least 3 months after the initial CT scan and no sooner than 3 days before surgery.

### Protein Expression Classifier Analysis

Samples collected from subjects prior to definitive excision or treatment of the nodule were analyzed using multiple reaction monitoring as previously described [6, 8]. The protein classifier, Xpresys^®^ Lung, consists of 5 diagnostic and 6 normalization proteins and has been analytically and clinically validated [6, 8, 9]. The classifier reports “likely benign” when the probability of the lung nodule having benign etiology is high (NPV at least 84 %) and the remainder are reported as “indeterminate.” In accordance with best practices, the classifier was adjusted for evaluation of archival specimens included in this study (see Supplementary). Physicians and patients were blinded to the protein classifier results and laboratory personnel blinded to clinical outcomes.

### Statistical Analysis

To assess the ability of the classifier to improve the diagnostic evaluation of indeterminate lung nodules and its clinical utility, we assessed all procedures and outcomes and modeled the change in invasive procedures that would occur if the classifier results were used prospectively for decision making. The primary outcomes for this analysis were the potential benefits, i.e., reduction in invasive procedures (TTNA, bronchoscopy, and surgery), and the potential harms, i.e., rate of patients with malignant nodules routed to CT surveillance. This analysis assumes that all patients with a likely benign result would not undergo surgery or other invasive testing, but instead would be evaluated by CT surveillance.

Continuous and categorical variables were assessed using Mann–Whitney and Fisher’s exact tests. Confidence intervals (CIs) are reported as two-sided binomial 95 % CIs. Statistical analysis was performed using the R statistical package [10].

## Results

**Fig. 1:**
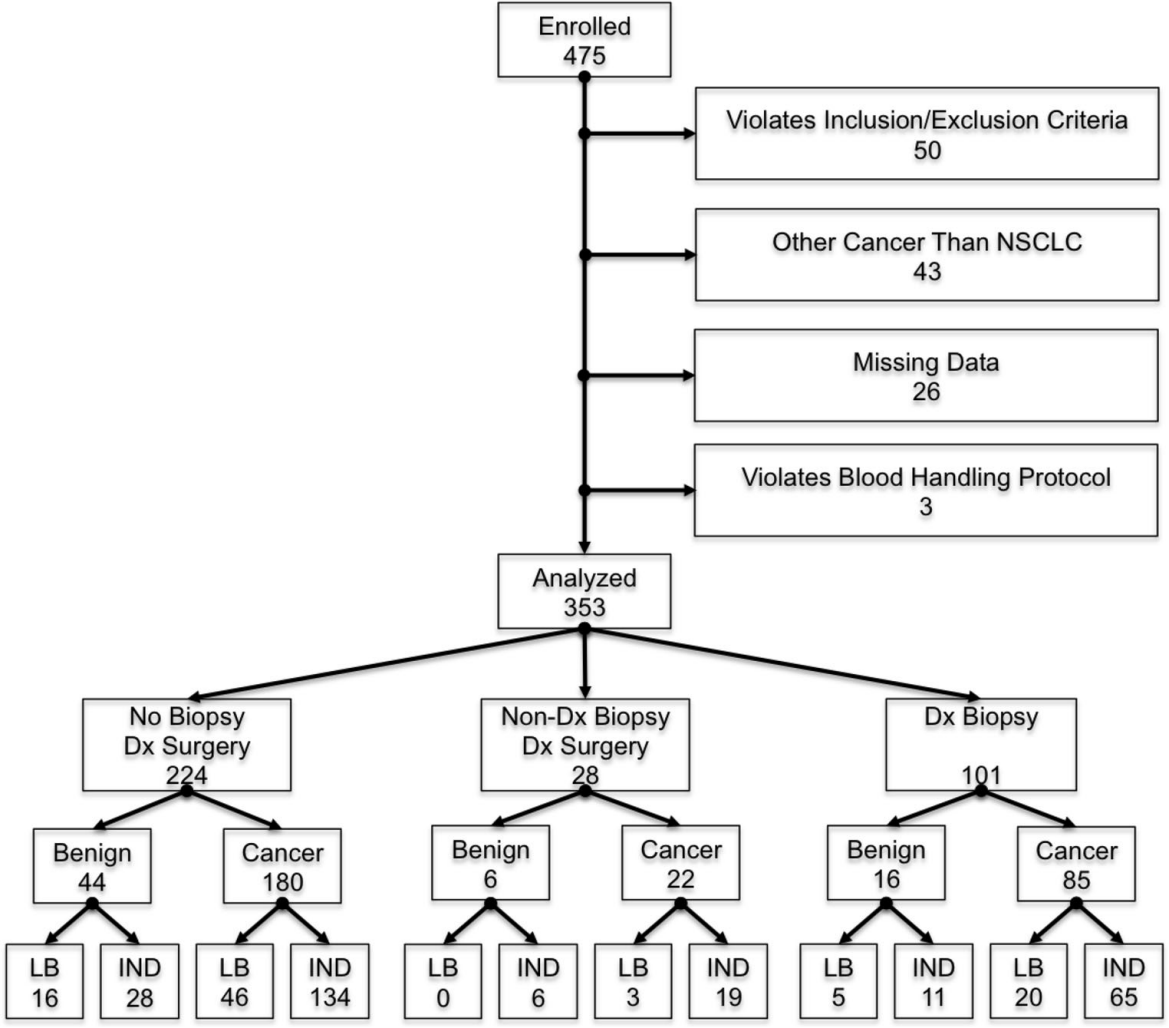
Flowchart of subjects included in clinical utility analyses along with categorization of subjects by procedure, outcome and classifier report. ‘LB’ and ‘IND’ represent a classifier ‘Likely Benign’ and ‘Indeterminate’ report, respectively. Dx indicates diagnostic, Non-Dx is a non-diagnostic procedure, and Dx Biopsy includes patients with a biopsy only and those that had a diagnostic biopsy followed by surgery

A total of 475 subjects were enrolled prospectively from April 2012 to December 2014 in the registered study, NCT01752101. Of these, 353 patients were eligible for the clinical utility analysis; 287 (81.3 %, CI 76.8–85.2 %) were diagnosed with NSCLC, and 66 (18.7 %, CI 14.8–23.2 %) diagnosed with a benign lung nodule (Fig. 1). Reasons for subject ineligibility included violating the inclusion/exclusion requirements of the registered study (50 subjects), diagnosed with a cancer other than NSCLC (43 subjects), unable to obtain a definitive diagnosis due to missing data (26 subjects) and improper sample handling resulting in unanalyzable sample (3 subjects). Baseline demographics are shown in Table 1. Lung cancer patients were older (*p* value 0.03), and had greater cumulative tobacco pack-years (*p* value 0.04). No difference in nodule size between groups was observed (*p* value 0.45).

**Table 1:** Patient demographics stratified by diagnosis

Characteristics	All patients	Cancer	Benign	*p* value
Patients	353	287	66	
Age (years) [mean (range)]	68 (44.7–95.5)	68.6 (45.3–95.5)	65.4 (44.7–87.5)	0.027^a^
Gender (*n*, %)				0.272^b^
Male	154 (43.6)	121 (42.2)	33 (50.0)	
Female	199 (56.4)	166 (57.8)	33 (50.0)	
Smoking history				
Status (*n*, %)				0.220^b^
Never	60 (17.0)	46 (16.0)	14 (21.2)	
Former	210 (59.5)	173 (60.2)	37 (56.1)	
Current	76 (21.5)	64 (22.3)	12 (18.2)	
Passive exposure	7 (2.0)	4 (1.3)	3 (4.5)	
Pack-year mean (range)^c^	41.5 (0.5–150)	42.8 (0.8–150)	35.1 (0.5–120)	0.044^a^
Lung nodules				
Size (mm) [mean (range)]	18.5 (8.0–30)	18.6 (8.0–30)	18.0 (8.0–30)	0.447^a^

^a^Based on Mann–Whitney test (cancer versus benign)

^b^Based on Fisher’s exact test (cancer versus benign)

^c^Based on *n* = 285 (benign = 48 and cancer = 237)

The 353 patients were evaluated by procedure type (Fig. 1). A total of 101 patients were enrolled following a diagnostic TTNA or bronchoscopy biopsy, yielding 16 (15.8 %, CI 9.3–24.4 %) benign lesions and 85 (84.2 %, 75.6–90.7 %) patients with NSCLC. A total of 252 patients underwent a surgical lung biopsy; 224 (88.9 %, CI 84.3–92.5 %) did not have a preceding biopsy and 28 (11.1 %, CI 7.5–15.7 %) were performed following a biopsy that was non-diagnostic. Among the 224 patients proceeding directly to surgical lung biopsy, 44 had a benign diagnosis (19.6 %, CI 14.7-25.5 %) and 180 had NSCLC (80.4 %, CI 74.5–85.3 %). Among the 28 patients undergoing surgical lung biopsy following a non-diagnostic biopsy, 6 had a benign diagnosis (21.4 %, CI 8.3–41 %) and 22 had NSCLC (78.6 %, CI 59–91.7 %).

In total, 66 of 353 (18.7 %, CI 14.8–23.2 %) patients that underwent an invasive procedure (TTNA, bronchoscopy, surgical lung biopsy) were ultimately diagnosed with a benign nodule. This included 50 of 252 (19.8 %, CI 15.1–25.3 %) patients that underwent a surgical lung biopsy and 22 of 129 (17.1 %, CI 11.0–24.7 %) patients that had a bronchoscopy or TTNA. Six (9.1 %, CI 3.4–18.7 %) patients with benign lesions underwent multiple invasive procedures.

To estimate the effect of the classifier on the number of invasive procedures in patients with benign nodules, we determined the “likely benign” classifier result among patients. The classifier predicted the nodule to be likely benign in 16 of 50 patients (32.0 %. CI 19.5–46.7 %) determined to have a benign lesion by surgical lung biopsy. When considering all invasive procedures, the classifier predicted the nodule to be likely benign in 21 of 66 patients (31.8 %, CI 20.9–44.4 %) with benign nodules diagnosed by either TTNA, bronchoscopy biopsy, or surgical lung biopsy.

Of the 287 cancers identified in the study, the classifier predicted “likely benign” in 49 patients (17.1 %, CI 12.9–21.9 %) that were diagnosed by surgical biopsy and in 69 patients (24.0 %, CI 19.2–29.4 %) diagnosed by any invasive procedure. Consequently, 17–24 % of patients with a lung cancer nodule would be triaged to CT surveillance. This was similar to the rate observed in the retrospective-observational cohort where 23 of 94 patients (24.5 %, CI 16.2–34.4 %) with nodules ultimately diagnosed as NSCLC underwent CT surveillance during nodule management.

## Discussion

This study presents the clinical utility of a protein classifier for the management of indeterminate pulmonary nodules. Clinical utility is “the balance of benefits and harms associated with the use of the test in practice, including improvement in measureable clinical outcomes and the usefulness or added value in decision-making compared with not using the test” [11]. This prospective–retrospective study demonstrates that use of the classifier produces the potential benefit of reducing unnecessary surgeries (32.0 %) and invasive procedures (31.8 %). The potential harm (malignant lung nodules routed to CT surveillance) is 17.1 % if the classifier is used prior to surgery and 24.0 % if used prior to any invasive procedure. This compares favorably to 24.5 % as observed in 18 pulmonary clinic practices [3, 4]. This indicates that this classifier can provide incremental clinical utility over usual care. Importantly, the level of evidence is high at level 1B [12] which compares favorably to the GRADE 1C and 2C recommendations within the American College of Chest Physicians (ACCP) guidelines on pulmonary nodule management [13–15].

The risks and expense of biopsies and surgery are considerable with TTNA having pneumothorax rates of 15 % and surgery mortality rates of 1–5 % [13]. Notably, nearly 10 % of patients in this study had multiple procedures. These rates of complications are of increasing concern as screening LDCT is now recommended for those at high risk of lung cancer. Thirty-nine percent of patients undergoing LDCT had a least one positive screen, with the majority (96 %) being false positives [16].

The strengths of this study include its magnitude, geographic diversity, high level of evidence, and generalizability. A limitation is the comparison of rates of malignant nodules routed to CT surveillance by usual care (24.5 %) to the classifier (17.1 %; 24.0 %). Although both estimates are taken over all malignant nodules, the former includes nodules between 8 and 20 mm and the present analysis includes nodules between 8 and 30 mm in diameter.

**Fig. 2:**
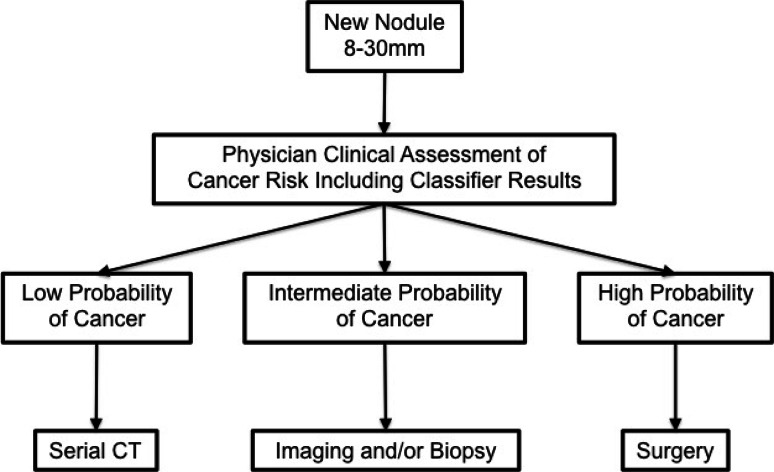
The incorporation of the classifier into the ACCP guidelines for lung nodule management. Newly identified lung nodules between 8 and 30 mm in diameter are assessed using the plasma protein classifier. The classifier results are integrated into the physician’s assessment of cancer risk

In summary, this classifier can assist physicians with the challenging task of differentiating benign and malignant pulmonary nodules for the purpose of reducing unnecessary invasive procedures on benign nodules. This classifier can be used early in the evaluation process, in combination with physician assessment, and before choosing an invasive procedure in the evaluation of newly discovered 8–30 mm pulmonary nodules (Fig. 2).

## Electronic supplementary material

Supplementary material 1 (PDF 43 kb)

Supplementary material 2 (PDF 65 kb)
