# Supplementary material for: Clinical Utility of a Plasma Protein Classifier for Indeterminate Lung Nodules
Source: Lung. 2015 Sep 16;193(6):1023–7. doi: 10.1007/s00408-015-9800-0 (PMC4651976; doi:10.1007/s00408-015-9800-0)
Supplement: Supplementary file 1 — Supplementary material 1 (PDF 43 kb) [file 408_2015_9800_MOESM1_ESM.pdf]

Title:
Clinical Utility of a Plasma Protein Classifier for Indeterminate Lung Nodules

Journal:
Lung

Authors:
Anil Vachani[1]; Zane Hammoud[2]; Steven Springmeyer[7]; Neri Cohen[3]; Dao Nguyen[4]; Christina Williamson[5]; Sandra Starnes[6]; Stephen Hunsucker[7]; Scott Law[7]; Xiao-Jun Li[7]; Alexander Porter[7]; Paul Kearney[7]

[1] Pulmonary, Allergy, and Critical Care Division, Perelman School of Medicine, University of Pennsylvania/Abramson Research Center, 3615 Civic Center Boulevard, Suite 1016E, Philadelphia, PA 19104
[2]Henry Ford Hospital, 2799 W. Grand Blvd., Detroit, MI 48202
[3]Greater Baltimore Medical Center, 6569 North Charles Street, Suite 701, Baltimore, MD 21204
[4]Sylvester Comprehensive Cancer Center, University of Miami Hospital & Clinics, 1550 NW 10th Avenue, Fox Building, Suite 308, Office 314, Miami, FL 33136
[5]Lahey Hospital & Medical Center, Department of Thoracic and Cardiovascular Surgery, 41 Mall Road, Burlington, MA 01805
[6]University of Cincinnati, 231 Albert Sabin Way, Cincinnati, OH 45267-0562
[7]Integerated Diagnostics, 818 Stewart St., Suite 1101, Seattle, WA, 98101

Address Correspondence:
Paul Kearney, Ph.D.
Integrated Diagnostics
818 Stewart St., Suite 1101
Seattle, WA 98101
Email: pkearney@indidx.com
Phone: 206-576-6311
Fax: 206-576-6350

SUPPLEMENTARY

Archival Version of the Protein Classifier

Best practices for analysis of archival samples by an assay requires that an archival version of the assay be developed and assessed as measured analytes can change with storage time [11]. The protein classifier consists of 5 diagnostic proteins and 6 normalizing proteins. Two adjustments were made to the protein classifier as described below.

First, to account for protein-specific storage time effects, the expression of each diagnostic protein was adjusted by a pre-factor (ALDOA, 2.2469; COIA1, 1.0581; FRIL, 1.9598; LG3BP, 1.2056; and TSP1, 2.6871). Pre-factors were derived based on observed changes in the range of protein measurements.

Second, to account for different storage times for patient samples (samples were collected over a 33 month enrolment period), a storage time dependent decision threshold was incorporated as a parameter into the classification model. This storage time dependent decision threshold was derived and applied as follows:
Fifty benign samples and 50 matching cancer samples were randomly selected. Each sample pair was from the same collection site, had similar length of storage time (within 44 days), and were from patients having lung nodules of similar size (median difference of 3mm). The 50 sample pairs were then evenly divided into two groups based on their average storage time: Group A contained the older samples (> 254 days) and Group B contained the younger samples (≤ 254 days) where 254 days was the median storage time separating Groups A and B. 10,000 bootstrapping simulations were used to determine a threshold (0.3253) from samples in Group A: In each simulation, a threshold that corresponded to a negative predictive value of 84% was determined from 20 randomly selected benign/cancer sample pairs. When necessary, the method of linear interpolation was used. The threshold for Group A was the corresponding average of all the thresholds from the simulations. Similarly, a threshold (0.4486) was determined from samples in Group B. Consequently, all 353 samples analyzed in the study used either the Group A decision threshold (if over 254 days stored) or the Group B decision threshold (if 254 or fewer days stored) to determine the classification into “Likely Benign” or “Indeterminate”. The selection and derivation of these storage age thresholds was performed by an external consultant keeping the internal analysis team blinded.
