# Supplementary material for: Clinical Utility of a Plasma Protein Classifier for Indeterminate Lung Nodules
Source: Lung. 2015 Sep 16;193(6):1023–7. doi: 10.1007/s00408-015-9800-0 (PMC4651976; doi:10.1007/s00408-015-9800-0)
Supplement: Supplementary file 2 — Supplementary material 2 (PDF 65 kb) [file 408_2015_9800_MOESM2_ESM.pdf]

Title Page

Clinical Utility of a Plasma Protein Classifier for Indeterminate Lung Nodules

Anil Vachani1; Zane Hammoud2; Steven Springmeyer7; Neri Cohen3; Dao Nguyen4; Christina Williamson5; Sandra Starnes6; Stephen Hunsucker7; Scott Law7; Xiao-Jun Li7; Alexander Porter7; Paul Kearney7

1 Pulmonary, Allergy, and Critical Care Division, Perelman School of Medicine, University of Pennsylvania/Abramson Research Center, 3615 Civic Center Boulevard, Suite 1016E, Philadelphia, PA 19104
2Henry Ford Hospital, 2799 W. Grand Blvd., Detroit, MI 48202
3Greater Baltimore Medical Center, 6569 North Charles Street, Suite 701, Baltimore, MD 21204
4Sylvester Comprehensive Cancer Center, University of Miami Hospital & Clinics, 1550 NW 10th Avenue, Fox Building, Suite 308, Office 314, Miami, FL 33136
5Lahey Hospital & Medical Center, Department of Thoracic and Cardiovascular Surgery, 41 Mall Road, Burlington, MA 01805
6University of Cincinnati, 231 Albert Sabin Way, Cincinnati, OH 45267-0562
7Integrated Diagnostics, 818 Stewart St., Suite 1101, Seattle, WA, 98101

Address Correspondence:
Paul Kearney, Ph.D.
Integrated Diagnostics
818 Stewart St., Suite 1101
Seattle, WA 98101
Email: pkearney@indidx.com
Phone: 206-576-6311
Fax: 206-576-6350

Age	Sex	Smoking	Pack-	Nodu	Adjudicated Pro	Primary	Xpresys Result
74	Male	Former	60	12	Surgery Only	NSCLC	Indeterminate
71	Female	Never	NA	25	Surgery Only	NSCLC	Likely Benign
68	Male	Current	42	22	Surgery Only	NSCLC	Indeterminate
74	Female	Never	NA	30	Biopsy Only	NSCLC	Likely Benign
66	Female	Former	40	24	Biopsy Only	Benign	Indeterminate
67	Male	Former	71	27	Biopsy & Surger	NSCLC	Indeterminate
81	Male	Current	47	21	Biopsy Only	Benign	Indeterminate
62	Female	Former	20	20	Surgery Only	NSCLC	Indeterminate
63	Female	Never	NA	27	Biopsy & Surger	NSCLC	Indeterminate
66	Male	Never	NA	10	Surgery Only	Benign	Likely Benign
76	Female	Former	69	20	Biopsy & Surger	NSCLC	Likely Benign
75	Male	Former	57	20	Biopsy & Surger	NSCLC	Indeterminate
76	Female	Former	22	27	Biopsy & Surger	NSCLC	Indeterminate
82	Female	Never	NA	16	Biopsy Only	NSCLC	Indeterminate
81	Female	Never	NA	20	Biopsy & Surger	NSCLC	Indeterminate
79	Male	Never	NA	30	Surgery Only	NSCLC	Indeterminate
58	Female	Former	20	8	Biopsy & Surger	NSCLC	Indeterminate
81	Male	Never	NA	20	Biopsy Only	Benign	Likely Benign
65	Female	Never	NA	27	Biopsy & Surger	NSCLC	Indeterminate
65	Male	Former	43	12	Biopsy Only	NSCLC	Indeterminate
73	Female	Never	NA	26	Surgery Only	NSCLC	Indeterminate
68	Female	Former	25	24	Surgery Only	NSCLC	Indeterminate
58	Female	Never	NA	29	Biopsy & Surger	NSCLC	Indeterminate
69	Female	Current	27	28	Biopsy Only	Benign	Likely Benign
68	Female	Current	20	12	Biopsy & Surger	NSCLC	Indeterminate
72	Male	Former	40	24	Biopsy Only	NSCLC	Indeterminate
59	Female	Former	20	27	Surgery Only	NSCLC	Likely Benign
76	Male	Former	45	20	Surgery Only	NSCLC	Indeterminate
57	Male	Former	74	20	Biopsy & Surger	NSCLC	Indeterminate
77	Female	Former	11	18	Surgery Only	NSCLC	Indeterminate
82	Female	Never	NA	15	Surgery Only	NSCLC	Indeterminate
79	Female	Former	21	30	Surgery Only	Benign	Likely Benign
55	Female	Current	56	12	Surgery Only	NSCLC	Indeterminate
76	Female	Passive E	NA	14	Surgery Only	NSCLC	Indeterminate
74	Male	Former	30	28	Biopsy Only	NSCLC	Indeterminate
75	Male	Former	40	26	Surgery Only	NSCLC	Indeterminate
65	Female	Former	20	15	Surgery Only	NSCLC	Indeterminate
77	Male	Former	35	8	Surgery Only	NSCLC	Indeterminate
65	Female	Never	NA	30	Surgery Only	NSCLC	Indeterminate
77	Female	Former	80	18	Surgery Only	NSCLC	Indeterminate

64	Male	Current	75	21	Biopsy Only	NSCLC	Indeterminate
84	Male	Former	36	27	Surgery Only	NSCLC	Indeterminate
60	Male	Former	70	20	Surgery Only	NSCLC	Likely Benign
64	Female	Passive E	NA	12	Biopsy Only	Benign	Likely Benign
63	Male	Never	NA	21	Biopsy Only	Benign	Likely Benign
66	Female	Current	40	11	Surgery Only	NSCLC	Indeterminate
65	Female	Never	NA	17	Surgery Only	NSCLC	Indeterminate
48	Male	Former	30	8	Surgery Only	Benign	Likely Benign
70	Female	Former	52	14	Surgery Only	NSCLC	Indeterminate
72	Male	Former	8	12	Surgery Only	NSCLC	Indeterminate
54	Female	Never	NA	11	Surgery Only	NSCLC	Indeterminate
91	Male	Former	70	27	Biopsy Only	NSCLC	Indeterminate
65	Female	Former	40	20	Surgery Only	NSCLC	Indeterminate
58	Female	Former	20	22	Surgery Only	NSCLC	Indeterminate
69	Male	Former	20	14	Surgery Only	NSCLC	Indeterminate
71	Female	Former	51	28	Surgery Only	NSCLC	Likely Benign
70	Female	Former	4	13	Surgery Only	NSCLC	Indeterminate
61	Male	Never	NA	11	Surgery Only	Benign	Likely Benign
73	Female	Never	NA	15	Surgery Only	NSCLC	Likely Benign
52	Male	Never	NA	23	Surgery Only	Benign	Indeterminate
71	Female	Former	45	20	Surgery Only	NSCLC	Indeterminate
76	Male	Former	50	14	Surgery Only	NSCLC	Likely Benign
72	Male	Former	10	14	Surgery Only	Benign	Indeterminate
59	Male	Current	60	16	Surgery Only	NSCLC	Indeterminate
49	Female	Never	NA	16	Surgery Only	NSCLC	Likely Benign
64	Female	Former	14	8	Surgery Only	NSCLC	Indeterminate
63	Male	Former	50	14	Surgery Only	Benign	Likely Benign
83	Female	Former	5	16	Surgery Only	Benign	Indeterminate
73	Male	Former	60	21	Surgery Only	NSCLC	Indeterminate
61	Male	Never	NA	25	Surgery Only	NSCLC	Indeterminate
72	Female	Never	NA	18	Surgery Only	NSCLC	Indeterminate
68	Male	Former	18	24	Surgery Only	NSCLC	Indeterminate
86	Male	Former	60	18	Surgery Only	NSCLC	Indeterminate
59	Female	Never	NA	20	Surgery Only	NSCLC	Likely Benign
65	Male	Former	23	22	Biopsy Only	NSCLC	Likely Benign
56	Male	Former	40	10	Surgery Only	NSCLC	Indeterminate
76	Male	Former	80	20	Biopsy Only	NSCLC	Likely Benign
83	Male	Former	40	23	Surgery Only	NSCLC	Likely Benign
79	Female	Former	20	28	Surgery Only	NSCLC	Likely Benign
79	Male	Former	45	20	Surgery Only	NSCLC	Indeterminate
66	Female	Former	40	28	Surgery Only	NSCLC	Likely Benign
83	Male	Former	45	16	Surgery Only	Benign	Indeterminate

71	Male	Former	5	18	Surgery Only	NSCLC	Indeterminate
78	Female	Current	50	23	Biopsy Only	NSCLC	Indeterminate
64	Male	Former	60	10	Biopsy Only	NSCLC	Indeterminate
74	Female	Former	20	11	Biopsy Only	Benign	Indeterminate
66	Male	Former	45	20	Surgery Only	NSCLC	Indeterminate
67	Female	Current	75	20	Surgery Only	NSCLC	Indeterminate
56	Female	Former	45	19	Surgery Only	NSCLC	Indeterminate
73	Male	Former	90	22	Surgery Only	NSCLC	Indeterminate
77	Female	Former	10	15	Surgery Only	NSCLC	Indeterminate
73	Female	Former	84	12	Surgery Only	NSCLC	Indeterminate
56	Female	Never	NA	16	Surgery Only	NSCLC	Indeterminate
80	Female	Former	20	24	Biopsy Only	NSCLC	Indeterminate
73	Female	Former	20	24	Surgery Only	NSCLC	Indeterminate
70	Female	Never	NA	13	Surgery Only	NSCLC	Indeterminate
52	Female	Former	10	13	Surgery Only	Benign	Indeterminate
70	Female	Former	45	17	Surgery Only	NSCLC	Indeterminate
70	Female	Former	45	17	Surgery Only	NSCLC	Indeterminate
70	Male	Former	64	16	Surgery Only	NSCLC	Likely Benign
76	Male	Former	83	16	Biopsy Only	NSCLC	Indeterminate
76	Male	Former	60	26	Surgery Only	NSCLC	Indeterminate
65	Female	Never	NA	9	Surgery Only	Benign	Indeterminate
70	Female	Never	NA	12	Surgery Only	NSCLC	Indeterminate
65	Male	Former	100	30	Surgery Only	NSCLC	Indeterminate
62	Male	Former	56	23	Surgery Only	NSCLC	Indeterminate
70	Male	Former	30	23	Surgery Only	NSCLC	Likely Benign
81	Female	Former	20	15	Surgery Only	NSCLC	Likely Benign
57	Male	Current	40	10	Surgery Only	Benign	Indeterminate
72	Female	Former	60	27	Surgery Only	NSCLC	Likely Benign
72	Female	Current	40	18	Biopsy Only	Benign	Likely Benign
74	Male	Current	29	16	Biopsy Only	NSCLC	Indeterminate
56	Male	Current	20	17	Surgery Only	NSCLC	Indeterminate
61	Male	Former	70	18	Surgery Only	Benign	Likely Benign
71	Male	Never	NA	11	Surgery Only	NSCLC	Indeterminate
76	Female	Passive E	NA	13	Surgery Only	NSCLC	Indeterminate
77	Female	Former	25	23	Surgery Only	NSCLC	Likely Benign
68	Male	Former	10	20	Surgery Only	NSCLC	Likely Benign
69	Male	Current	50	20	Surgery Only	NSCLC	Indeterminate
68	Male	Former	75	18	Surgery Only	NSCLC	Likely Benign
66	Female	Never	NA	17	Surgery Only	NSCLC	Indeterminate
72	Male	Former	37	20	Surgery Only	NSCLC	Indeterminate
61	Female	Former	105	14	Surgery Only	NSCLC	Indeterminate
92	Male	Former	15	26	Surgery Only	NSCLC	Indeterminate

55	Female	Former	30	12	Biopsy Only	NSCLC	Indeterminate
67	Male	Former	80	18	Surgery Only	NSCLC	Likely Benign
71	Male	Former	80	16	Surgery Only	NSCLC	Likely Benign
70	Male	Former	NA	16	Surgery Only	Benign	Likely Benign
73	Female	Former	15	13	Biopsy Only	Benign	Indeterminate
68	Male	Former	115	22	Surgery Only	NSCLC	Indeterminate
59	Male	Former	20	30	Biopsy Only	NSCLC	Likely Benign
57	Female	Current	41	12	Surgery Only	NSCLC	Indeterminate
68	Female	Current	52	21	Surgery Only	Benign	Indeterminate
67	Male	Former	40	13	Surgery Only	NSCLC	Likely Benign
59	Male	Former	11	25	Surgery Only	NSCLC	Likely Benign
54	Female	Current	36	17	Surgery Only	NSCLC	Indeterminate
64	Male	Former	51	18	Surgery Only	NSCLC	Indeterminate
50	Female	Former	23	15	Biopsy & Surger	NSCLC	Indeterminate
68	Male	Former	28	17	Biopsy & Surger	NSCLC	Indeterminate
74	Female	Current	27	22	Biopsy & Surger	NSCLC	Indeterminate
72	Female	Former	36	11	Surgery Only	NSCLC	Indeterminate
60	Female	Current	43	11	Biopsy & Surger	NSCLC	Indeterminate
53	Female	Current	40	15	Surgery Only	NSCLC	Likely Benign
56	Female	Current	39	23	Surgery Only	NSCLC	Indeterminate
67	Male	Former	27	20	Surgery Only	NSCLC	Indeterminate
57	Female	Current	40	26	Biopsy & Surger	NSCLC	Indeterminate
71	Female	Passive E	NA	21	Surgery Only	NSCLC	Indeterminate
78	Female	Passive E	NA	11	Surgery Only	Benign	Indeterminate
47	Female	Current	15	22	Surgery Only	NSCLC	Indeterminate
58	Female	Former	26	13	Surgery Only	Benign	Indeterminate
89	Male	Former	50	22	Biopsy & Surger	NSCLC	Indeterminate
49	Female	Current	48	11	Biopsy & Surger	NSCLC	Indeterminate
61	Female	Former	45	13	Biopsy & Surger	NSCLC	Indeterminate
62	Female	Current	35	28	Biopsy Only	Benign	Indeterminate
82	Male	Former	80	24	Biopsy Only	Benign	Indeterminate
84	Male	Former	12	25	Biopsy & Surger	NSCLC	Indeterminate
65	Male	Former	44	22	Biopsy & Surger	NSCLC	Indeterminate
70	Male	Current	15	25	Biopsy & Surger	NSCLC	Indeterminate
67	Male	Current	46	13	Biopsy & Surger	NSCLC	Indeterminate
85	Male	Former	80	22	Surgery Only	NSCLC	Likely Benign
79	Female	Former	30	11	Biopsy & Surger	NSCLC	Likely Benign
69	Male	Former	30	14	Biopsy & Surger	NSCLC	Indeterminate
53	Male	Former	120	16	Biopsy Only	Benign	Indeterminate
53	Female	Current	14	23	Biopsy Only	NSCLC	Indeterminate
75	Male	Former	35	19	Biopsy & Surger	NSCLC	Likely Benign
84	Female	Former	40	22	Biopsy & Surger	NSCLC	Indeterminate

56	Female	Current	38	8	Surgery Only	NSCLC	Indeterminate
87	Male	Former	98	18	Biopsy Only	NSCLC	Indeterminate
69	Female	Former	21	24	Surgery Only	NSCLC	Indeterminate
65	Male	Former	32	9	Surgery Only	NSCLC	Indeterminate
59	Female	Current	42	13	Biopsy & Surger	NSCLC	Indeterminate
49	Female	Former	0.8	27	Biopsy & Surger	NSCLC	Indeterminate
72	Female	Former	40	8	Biopsy & Surger	NSCLC	Indeterminate
59	Female	Never	NA	11	Biopsy Only	Benign	Indeterminate
77	Male	Former	56	24	Biopsy Only	NSCLC	Indeterminate
73	Female	Current	31	22	Surgery Only	NSCLC	Indeterminate
67	Male	Current	50	18	Biopsy Only	Benign	Indeterminate
82	Male	Former	56	25	Biopsy & Surger	NSCLC	Likely Benign
88	Female	Former	1.5	26	Surgery Only	NSCLC	Indeterminate
60	Female	Former	41	13	Surgery Only	NSCLC	Likely Benign
90	Female	Never	NA	12	Biopsy Only	NSCLC	Likely Benign
81	Female	Never	NA	30	Biopsy & Surger	NSCLC	Indeterminate
51	Female	Current	35	13	Surgery Only	Benign	Likely Benign
77	Female	Never	NA	25	Biopsy & Surger	NSCLC	Likely Benign
59	Female	Never	NA	13	Biopsy Only	NSCLC	Likely Benign
52	Female	Former	40	20	Biopsy & Surger	NSCLC	Indeterminate
69	Female	Former	55	13	Surgery Only	NSCLC	Indeterminate
78	Female	Current	60	21	Biopsy & Surger	NSCLC	Indeterminate
80	Female	Former	40	11	Biopsy & Surger	NSCLC	Indeterminate
72	Male	Former	47	14	Biopsy & Surger	NSCLC	Indeterminate
77	Male	Former	65	15	Biopsy & Surger	Benign	Indeterminate
63	Male	Former	25	28	Surgery Only	Benign	Indeterminate
49	Female	Former	20	12	Surgery Only	Benign	Likely Benign
85	Male	Former	20	15	Surgery Only	NSCLC	Indeterminate
58	Male	Never	NA	13	Biopsy & Surger	NSCLC	Indeterminate
73	Male	Former	35	22	Biopsy & Surger	NSCLC	Indeterminate
73	Male	Former	60	13	Biopsy & Surger	NSCLC	Likely Benign
62	Female	Never	NA	27	Surgery Only	Benign	Likely Benign
57	Female	Former	25	18	Surgery Only	NSCLC	Likely Benign
81	Female	Former	40	8	Surgery Only	NSCLC	Likely Benign
77	Male	Former	80	14	Biopsy & Surger	NSCLC	Indeterminate
61	Male	Current	19	18	Biopsy & Surger	NSCLC	Indeterminate
83	Male	Former	15	15	Surgery Only	NSCLC	Likely Benign
56	Female	Former	30	10	Surgery Only	NSCLC	Indeterminate
68	Female	Never	NA	14	Surgery Only	NSCLC	Likely Benign
66	Female	Former	44	10	Surgery Only	NSCLC	Indeterminate
62	Female	Never	NA	17	Surgery Only	NSCLC	Indeterminate
72	Male	Former	63	22	Surgery Only	NSCLC	Likely Benign

63	Male	Former	36	14	Biopsy & Surger	NSCLC	Indeterminate
66	Male	Current	30	23	Biopsy & Surger	NSCLC	Indeterminate
50	Female	Current	20	12	Surgery Only	NSCLC	Indeterminate
61	Male	Former	60	16	Biopsy & Surger	Benign	Indeterminate
72	Female	Current	74	17	Biopsy Only	NSCLC	Indeterminate
70	Male	Former	18	17	Biopsy & Surger	NSCLC	Indeterminate
50	Female	Current	120	20	Biopsy & Surger	NSCLC	Indeterminate
67	Female	Passive E	NA	20	Surgery Only	Benign	Indeterminate
71	Male	Current	54	28	Surgery Only	NSCLC	Indeterminate
72	Female	Former	10	18	Biopsy & Surger	NSCLC	Likely Benign
49	Male	Current	15	20	Biopsy & Surger	Benign	Indeterminate
81	Female	Passive E	NA	18	Surgery Only	NSCLC	Likely Benign
73	Male	Former	10	11	Surgery Only	NSCLC	Indeterminate
78	Female	Former	40	28	Surgery Only	NSCLC	Indeterminate
50	Female	Current	20	12	Surgery Only	NSCLC	Indeterminate
79	Female	Never	NA	23	Biopsy & Surger	NSCLC	Indeterminate
70	Female	Former	20	14	Biopsy & Surger	NSCLC	Indeterminate
84	Female	Former	35	22	Surgery Only	Benign	Indeterminate
87	Male	Former	20	22	Biopsy Only	NSCLC	Indeterminate
53	Male	Current	20	22	Biopsy & Surger	NSCLC	Indeterminate
72	Male	Former	68	24	Surgery Only	Benign	Likely Benign
60	Female	Former	4.5	22	Biopsy & Surger	NSCLC	Indeterminate
53	Female	Former	60	11	Surgery Only	NSCLC	Indeterminate
69	Male	Former	50	25	Biopsy & Surger	NSCLC	Indeterminate
84	Male	Current	60	22	Surgery Only	NSCLC	Indeterminate
49	Female	Former	25	20	Biopsy & Surger	NSCLC	Indeterminate
63	Male	Current	25	27	Surgery Only	NSCLC	Indeterminate
53	Male	Current	10	26	Biopsy & Surger	Benign	Indeterminate
56	Female	Current	24	21	Surgery Only	NSCLC	Indeterminate
60	Female	Former	37	14	Biopsy & Surger	Benign	Indeterminate
68	Male	Never	NA	29	Surgery Only	Benign	Indeterminate
89	Female	Former	8	28	Biopsy Only	NSCLC	Indeterminate
76	Male	Current	48	12	Surgery Only	NSCLC	Indeterminate
85	Male	Current	38	12	Surgery Only	NSCLC	Indeterminate
74	Female	Former	1.5	28	Surgery Only	NSCLC	Indeterminate
67	Male	Former	15	8	Surgery Only	Benign	Likely Benign
69	Female	Current	105	11	Surgery Only	NSCLC	Indeterminate
76	Male	Current	30	23	Surgery Only	NSCLC	Likely Benign
60	Female	Former	30	27	Biopsy & Surger	NSCLC	Likely Benign
65	Female	Never	NA	29	Surgery Only	NSCLC	Indeterminate
69	Female	Former	80	29	Surgery Only	NSCLC	Indeterminate
85	Female	Current	89	13	Biopsy & Surger	NSCLC	Indeterminate

69	Female	Never	NA	25	Surgery Only	Benign	Likely Benign
83	Male	Former	32	24	Biopsy & Surger	NSCLC	Indeterminate
54	Female	Former	9.5	20	Surgery Only	NSCLC	Indeterminate
63	Female	Former	50	27	Biopsy & Surger	NSCLC	Indeterminate
75	Female	Never	NA	21	Surgery Only	NSCLC	Indeterminate
60	Female	Former	43	8	Surgery Only	NSCLC	Likely Benign
71	Female	Former	25	21	Biopsy & Surger	NSCLC	Likely Benign
66	Female	Former	45	15	Biopsy & Surger	NSCLC	Indeterminate
63	Female	Current	82	17	Biopsy & Surger	NSCLC	Indeterminate
50	Female	Former	70	25	Surgery Only	Benign	Likely Benign
66	Male	Current	45	28	Surgery Only	NSCLC	Indeterminate
71	Male	Current	38	13	Surgery Only	Benign	Indeterminate
80	Male	Former	50	16	Surgery Only	NSCLC	Indeterminate
65	Male	Current	25	23	Surgery Only	NSCLC	Indeterminate
64	Female	Former	40	30	Surgery Only	NSCLC	Indeterminate
90	Female	Never	NA	29	Surgery Only	NSCLC	Indeterminate
63	Female	Former	20	15	Surgery Only	NSCLC	Indeterminate
65	Male	Current	138	17	Surgery Only	NSCLC	Likely Benign
71	Female	Former	30	22	Surgery Only	Benign	Likely Benign
51	Female	Former	16	15	Surgery Only	NSCLC	Likely Benign
76	Male	Former	45	24	Surgery Only	NSCLC	Indeterminate
72	Male	Former	45	18	Biopsy Only	NSCLC	Likely Benign
66	Male	Former	40	15	Surgery Only	NSCLC	Indeterminate
63	Female	Current	30	17	Surgery Only	NSCLC	Indeterminate
64	Male	Current	90	12	Surgery Only	NSCLC	Indeterminate
59	Female	Current	52	14	Surgery Only	NSCLC	Likely Benign
75	Female	Former	20	10	Biopsy Only	NSCLC	Likely Benign
51	Female	Former	76	14	Biopsy & Surger	NSCLC	Indeterminate
56	Male	Current	15	28	Biopsy & Surger	NSCLC	Indeterminate
54	Female	Current	42	15	Biopsy & Surger	NSCLC	Likely Benign
56	Male	Never	NA	28	Surgery Only	NSCLC	Indeterminate
75	Female	Former	40	18	Biopsy & Surger	NSCLC	Indeterminate
79	Male	Former	1.5	14	Surgery Only	Benign	Indeterminate
74	Female	Former	7	13	Surgery Only	NSCLC	Indeterminate
66	Female	Former	47	17	Biopsy & Surger	NSCLC	Indeterminate
81	Female	Former	50	25	Surgery Only	NSCLC	Indeterminate
64	Female	Current	50	14	Surgery Only	NSCLC	Likely Benign
76	Male	Current	61	14	Surgery Only	NSCLC	Indeterminate
79	Female	Current	60	26	Surgery Only	NSCLC	Indeterminate
66	Male	Former	80	14	Surgery Only	NSCLC	Indeterminate
95	Male	Former	150	19	Biopsy & Surger	NSCLC	Likely Benign
56	Female	Never	NA	19	Biopsy & Surger	NSCLC	Indeterminate

68	Male	Former	45	24	Biopsy & Surger	NSCLC	Likely Benign
50	Male	Never	NA	30	Surgery Only	Benign	Indeterminate
56	Female	Current	40	11	Surgery Only	NSCLC	Indeterminate
70	Female	Current	55	11	Surgery Only	NSCLC	Indeterminate
53	Female	Current	60	23	Surgery Only	Benign	Likely Benign
64	Female	Never	NA	29	Biopsy & Surger	NSCLC	Indeterminate
66	Female	Former	11	14	Surgery Only	Benign	Indeterminate
60	Female	Current	30	13	Surgery Only	NSCLC	Indeterminate
67	Female	Current	106	16	Biopsy & Surger	NSCLC	Indeterminate
62	Male	Never	NA	23	Surgery Only	NSCLC	Indeterminate
79	Female	Former	25	25	Surgery Only	NSCLC	Indeterminate
71	Male	Current	80	13	Surgery Only	NSCLC	Indeterminate
84	Female	Former	10	22	Biopsy Only	Benign	Indeterminate
78	Female	Former	25	18	Surgery Only	NSCLC	Indeterminate
45	Male	Never	NA	15	Surgery Only	NSCLC	Likely Benign
58	Female	Former	40	20	Surgery Only	NSCLC	Indeterminate
54	Male	Former	15	20	Surgery Only	Benign	Indeterminate
55	Female	Former	40	9	Biopsy & Surger	NSCLC	Indeterminate
63	Female	Former	20	18	Biopsy & Surger	Benign	Indeterminate
54	Male	Former	14	30	Surgery Only	NSCLC	Indeterminate
66	Male	Former	25	19	Surgery Only	NSCLC	Likely Benign
61	Female	Current	45	14	Surgery Only	NSCLC	Indeterminate
64	Male	Former	60	10	Surgery Only	Benign	Indeterminate
52	Female	Former	15	9	Biopsy & Surger	NSCLC	Indeterminate
64	Female	Never	NA	13	Surgery Only	Benign	Indeterminate
51	Male	Former	25	19	Surgery Only	Benign	Indeterminate
60	Female	Former	10	15	Biopsy & Surger	NSCLC	Likely Benign
74	Male	Former	24	23	Biopsy Only	Benign	Likely Benign
66	Female	Former	38	10	Surgery Only	NSCLC	Indeterminate
56	Male	Former	15	11	Surgery Only	NSCLC	Likely Benign
73	Female	Former	30	11	Surgery Only	NSCLC	Likely Benign
70	Male	Former	50	13	Surgery Only	NSCLC	Likely Benign
58	Female	Former	30	19	Surgery Only	NSCLC	Likely Benign
69	Male	Former	40	27	Surgery Only	Benign	Indeterminate
72	Male	Former	80	22	Biopsy Only	NSCLC	Indeterminate
78	Female	Former	50	30	Surgery Only	NSCLC	Indeterminate
74	Female	Former	60	8	Surgery Only	NSCLC	Indeterminate
67	Female	Former	44	11	Surgery Only	NSCLC	Indeterminate
65	Male	Former	5	24	Surgery Only	NSCLC	Indeterminate
68	Female	Current	40	11	Surgery Only	NSCLC	Indeterminate
70	Female	Current	72	13	Biopsy & Surger	NSCLC	Indeterminate
77	Female	Former	13	14	Surgery Only	NSCLC	Indeterminate

69	Female	Never	NA	20	Biopsy & Surger	NSCLC	Indeterminate
75	Female	Former	25	15	Surgery Only	Benign	Indeterminate
69	Male	Former	75	25	Surgery Only	NSCLC	Indeterminate
76	Female	Former	71	14	Biopsy & Surger	NSCLC	Indeterminate
69	Female	Former	80	8	Surgery Only	NSCLC	Indeterminate
61	Male	Former	93	8	Surgery Only	NSCLC	Indeterminate
79	Male	Never	NA	16	Surgery Only	NSCLC	Likely Benign
74	Male	Former	27	11	Surgery Only	NSCLC	Likely Benign
63	Female	Never	NA	20	Biopsy & Surger	Benign	Indeterminate
87	Female	Never	NA	11	Surgery Only	Benign	Indeterminate
87	Male	Never	NA	23	Biopsy & Surger	NSCLC	Likely Benign
63	Female	Never	NA	22	Biopsy & Surger	NSCLC	Likely Benign
78	Female	Never	NA	13	Surgery Only	NSCLC	Indeterminate
64	Female	Never	NA	18	Surgery Only	NSCLC	Likely Benign
71	Female	Former	40	23	Surgery Only	Benign	Indeterminate
79	Male	Never	NA	30	Biopsy & Surger	NSCLC	Indeterminate
46	Female	Current	5	18	Surgery Only	NSCLC	Indeterminate
67	Female	Former	10	19	Surgery Only	NSCLC	Indeterminate
45	Male	Former	0.5	8	Surgery Only	Benign	Indeterminate
